# Increased Insulin Resistance and Hyperglycaemia in Long COVID: A Systematic Review and Meta-Analysis

**DOI:** 10.1017/erm.2026.10064

**Published:** 2026-07-07

**Authors:** Abbas F. Almulla, Yingqian Zhang, Chavit Tunvirachaisakul, Andre F. Carvalho, Michael Maes

**Affiliations:** 1 Sichuan Provincial Center for Mental Health, Sichuan Provincial People’s Hospital, School of Medicine, University of Electronic Science and Technology of China, Chengdu, 610072, China; 2 Key Laboratory of Psychosomatic Medicine, Chinese Academy of Medical Sciences, Chengdu, 610072, China; 3 Department of Psychiatry, Faculty of Medicine, Chulalongkorn University, Bangkok, Thailand; 4 Cognitive Impairment and Dementia Research Unit, Faculty of Medicine, Chulalongkorn University, Bangkok, Thailand; 5 Innovation in Mental and Physical Health and Clinical Treatment (IMPACT) Strategic Research Centre, School of Medicine, Barwon Health, Deakin University, Geelong, VIC, Australia; 6 Department of Psychiatry, Medical University of Plovdiv, Plovdiv, Bulgaria; 7 Research Institute, Medical University of Plovdiv, Plovdiv, Bulgaria; 8 Kyung Hee University, 26 Kyungheedae-ro, Dongdaemun-gu, Seoul 02447, Republic of Korea

**Keywords:** HbA1c, hyperglycaemia, insulin, insulin resistance, long COVID, metabolic disorder

## Abstract

**Background:**

Accumulating evidence suggests that long COVID (LC) is mediated by chronic immune activation, oxidative stress and metabolic dysregulation. These processes may impair glucose homeostasis and promote insulin resistance (IR). However, no prior meta-analysis has systematically and quantitatively evaluated IR indices and related biomarkers in LC compared with normal controls.

**Objectives:**

To systematically review and meta-analyse IR indices including HOMA-IR, HOMA-%B, HOMA-%S, insulin, fasting and random blood sugar (FBG, RBS), glycated haemoglobin (HbA1c), C-peptide and adipokine levels in individuals with LC compared with normal controls.

**Methods:**

PubMed, SCOPUS, SciFinder and Google Scholar databases were searched for relevant studies from inception to September 2025. Fifty-six eligible studies were included, comprising 9623 participants, 5340 LC patients and 4283 normal controls.

**Results:**

LC disease is characterized by elevated HOMA-IR (standardized mean difference, SMD = 0.539; 95% confidence interval, CI: 0.311; 0.766), insulin (SMD = 0.424; 95% CI: 0.113; 0.735), FBG and RBS (SMD = 0.734; 95% CI: 0.190; 1.277, SMD = 0.325; 95% CI: 0.133; 0.517). Furthermore, reduced HOMA-%S was observed in LC patients versus normal controls. Publication bias was generally low, though evidence of small-study effects was observed for several outcomes. The Immunological Confounder Scale was employed to evaluate study quality, revealing an overall intermediate methodological quality.

**Conclusions:**

Our results show that LC is associated with multiple abnormalities in glucose homeostasis, including increased HOMA-IR, hyperglycaemia and impaired insulin sensitivity indices, sustained metabolic disturbances beyond the acute phase.

## Introduction

A majority of individuals infected with coronavirus disease (COVID-19) experience recovery; however, a subset may develop long COVID (LC) condition, which can result in medium- to long-term effects influencing one or more organ systems (Ref. 1). The prevalence of this condition is reported to be as high as 36% and can reach up to 85% among hospitalized patients, depending on case definitions, sampling methodologies and follow-up duration (Refs 2–4). Clinical manifestations are diverse and affect multiple systems, with over 200 reported symptoms, including cardiovascular complications, metabolic disturbances, pancreatic involvement and frequent insulin resistance (IR) following the acute stage (Refs 5–7).

The pathophysiological mechanisms underpinning LC are not fully elucidated and may encompass immune dysregulation, oxidative stress and prolonged organ damage (Refs 8–12). LC is marked by heightened immune-inflammatory activity and oxidative and nitrosative stress (Refs 9, 13). A recent meta-analysis revealed activation of the immune-response system, M1 macrophage, T helper 1 and Th17 pathways, alongside elevated C-reactive protein concentrations (Ref. 9). In addition, aberrations in lipid metabolism, including reduced high-density lipoprotein (HDL) and apolipoprotein (Apo) A, and increased low-density lipoprotein (LDL), total cholesterol, ApoB and triglycerides have been reported in LC (Ref. 11). IR is closely linked to immune activation, dyslipidaemia and metabolic dysfunction, all of which have been implicated in LC (Refs 14–16). Adipokines, including adiponectin, leptin and ghrelin (Refs 17, 18), exhibited abnormal levels in LC and were significantly associated with IR (Ref. 19).

Acute COVID-19 markedly affects glucose metabolism via various mechanisms, resulting in hyperglycaemia and IR (Ref. 20). Severe acute respiratory syndrome coronavirus 2 (SARS-CoV-2) has a direct impact on pancreatic beta cells, essential for insulin synthesis (Ref. 21). The virus attaches to ACE2 receptors in the pancreas and other metabolic organs, which may lead to beta-cell dysfunction and reduced insulin secretion (Ref. 22). Harding et al. show that COVID-19 survivors exhibit increased susceptibility to developing new-onset diabetes mellitus (DM) (Ref. 23). Glycaemic abnormalities can continue after recovery, with changes in glucose metabolism and IR observed for up to 2 months following infection (Refs 7, 24). Additionally, numerous studies have indicated that LC is associated with increased fasting glucose, insulin levels and Homeostasis Model Assessment for Insulin Resistance (HOMA-IR), alongside diminished insulin sensitivity (Refs 7, 25, 26). IR is increasingly acknowledged as a factor in the clinical manifestations of LC including neuropsychiatric symptoms, such as fatigue, anxiety and depressive symptoms (Refs 7, 25, 27). The new-onset DM is likely attributable to direct injury to beta cells, systemic inflammation and the use of glucocorticoids during treatment, which exacerbate hyperglycaemia and IR (Ref. 20).

Although IR has been consistently observed in individuals diagnosed with LC, no meta-analysis has systematically aggregated and quantitatively assessed IR markers in LC patients compared to normal controls. Given the variability of the findings and sample sizes in individual evidence, quantitative synthesis by aggregating available studies facilitates the assessment of the extent and uniformity of metabolic irregularities and may enhance comprehension of their possible clinical implications. Therefore, this study examines IR indices in individuals with LC relative to controls. These include solitary biomarkers, comprise HOMA-IR, HOMA-%B, HOMA-%S, insulin, HbA1c, fasting blood glucose (FBG), random blood sugar (RBS) and C-peptide along with adipokines such as adiponectin, leptin and ghrelin. Furthermore, to comprehensively examine IR and glycaemic status in LC, composite measures including Glycaemic–Insulinaemic Dysregulation Index, Glucose–Insulin System Deviations, Insulin Secretion Axis Index and Integrated Glycaemic Burden were computed in this study. Based on this framework, we hypothesize that LC is associated with increased IR and hyperglycaemia.

## Materials and methods

This meta-analysis was designed and conducted in alignment with the Preferred Reporting Items for Systematic Reviews and Meta-Analyses (PRISMA 2020) statement (Ref. 28). Methodological decisions were additionally guided by the Cochrane Handbook for Systematic Reviews of Interventions (Ref. 29) and the recommendations from the Meta-analysis of Observational Studies in Epidemiology (MOOSE).

The study population comprised individuals with LC compared to control groups. We evaluated a broad spectrum of IR and glucose metabolism markers, including HOMA-IR, HOMA-%B, HOMA-%S, fasting insulin, HbA1c and C peptide. To provide a more comprehensive assessment, circulating levels of adipokines – notably adiponectin, leptin and ghrelin – were also examined. In addition to single biomarker analysis, we computed several composite indices that integrate multiple parameters of IR and β-cell function within the same eligible studies. These included the Glycaemic–Insulinaemic Dysregulation Index, Glucose–Insulin System Deviations, Insulin Secretion Axis Index and Integrated Glycaemic Burden.

This systematic review and meta-analysis was prospectively registered in the PROSPERO database under registration number CRD42025637597. Importantly, after protocol registration, various methodological enhancements were implemented, such as the extension of the search period to include newly published studies and the incorporation of additional exploratory outcomes to facilitate a more thorough evaluation of insulin resistance and glycaemic dysregulation in LC. These revisions did not change the principal purpose, study population, eligibility criteria or fundamental analytical methodology. Supplementary Table 1, displays a comprehensive comparison between the registered protocol and the final analysis.

### Search strategy

A systematic literature search was performed to identify studies reporting biomarkers of IR and glycaemia in LC patients. The search included primary electronic databases, namely PubMed/MEDLINE, Google Scholar, SciFinder and SCOPUS, spanning from inception to September 2025. Search queries were formulated using a combination of pre-defined keywords and Medical Subject Headings (MeSH) terms, as detailed in Supplementary Table 2. We also conducted a manual review of reference lists from eligible articles and relevant meta-analyses, thereby minimizing the risk of missing pertinent publications.

### Eligibility criteria

A thorough search was conducted to identify eligible studies, prioritizing peer-reviewed publications in English. However, grey literature and reports in other languages, such as Thai, French, Spanish, Turkish, Hungarian, German, Ukrainian, Italian, Russian and Arabic, were also included in the consideration. Searches were conducted using English-language search terms because the selected databases index most non-English publications through English titles, abstracts and keywords. The review team possessed proficiency in multiple languages, including Arabic, Chinese, Thai, Portuguese, Dutch, French and English, facilitating assessment of potentially eligible non-English studies. Furthermore, academic collaborators from other nations were consulted as required. As in our previous meta-analyses, studies were included if they employed an observational design (case–control or cohort), included an appropriate comparison group, examined IR or glucose homeostasis biomarkers in individuals with LC diagnosed according to WHO criteria (Ref. 30) and reported sufficient quantitative data for analysis. For longitudinal studies, only baseline biomarker assessments were considered.

The exclusion criteria included studies utilizing unconventional biological materials, such as hair, saliva, platelet-rich plasma or cerebrospinal fluid; studies limited to genetic or translational analyses; studies that did not include control groups and studies that lacked data on means with standard deviation (SD) or standard error (SE), unless these values were obtainable from the authors or could be calculated from published data using validated tools like the online calculator (https://www.math.hkbu.edu.hk/~tongt/papers/median2mean.html) (Ref. 31) and software (hkbu.edu.hk, https://automeris.io/WebPlotDigitizer/).

### Primary and secondary outcomes

This meta-analysis assessed various biomarkers indicative of IR including the key biomarkers namely HOMA-IR, HOMA-%B, HOMA-%S, insulin, FBG, HbA1c, RBS and C peptide as demonstrated in Table 1. Secondary analyses were conducted to examine the relevant adipokines such as adiponectin, leptin and ghrelin alongside composite indices reflective to insulin and glycaemic status in LC such as the Glycaemic–Insulinaemic Dysregulation Index, Glucose–Insulin System Deviations, Insulin Secretion Axis Index and Integrated Glycaemic Burden.

**Table 1.** The outcomes and number of patients with long COVID (LC) and normal controls along with the side of standardized mean difference (SMD) and the 95% confidence intervals with respect to zero SMD Table 1. long description.

Outcome profiles	*n* studies	Side of 95% confidence intervals				Patient cases	Control cases	Total number of participants
		<0	Overlap 0 and SMD <0	Overlap 0 and SMD >0	>0			
HOMA-IR	12	0	2	3	7	663	530	1193
Insulin	14	1	3	4	6	754	505	1259
Fasting blood glucose	12	1	1	5	5	887	754	1641
Random blood glucose	31	2	8	7	14	3424	2608	6032
HbA1c	16	1	3	6	6	1056	953	2009
HOMA-%B	5	1	0	2	2	269	232	501
HOMA-%S	2	2	0	0	0	146	69	215
C-peptide	3	0	1	3	0	109	306	415
Glycaemic–Insulinaemic Dysregulation index	7	1	0	2	4	551	351	902
Glucose–Insulin System Deviations	43	3	9	11	20	4316	3293	7609
Insulin Secretion Axis Index	3	0	0	3	0	50	63	113
Integrated Glycaemic Burden	49	3	9	14	23	4779	3830	8609
Adiponectin	4	0	1	3	0	125	166	291
Leptin	5	1	0	3	1	390	286	676
Ghrelin	3	1	0	1	1	78	62	140

HOMA-IR: homeostatic model assessment of insulin resistance; HbA1c: glycated haemoglobin; HOMA-%B: homeostatic model assessment of beta-cell function; HOMA-%S: homeostatic model assessment of insulin sensitivity.

### Screening and data extraction

Two investigators (AA and YZ) independently performed screening and data extraction, initially evaluating study titles and abstracts based on our pre-defined eligibility criteria. Full texts of potentially relevant articles were acquired for thorough evaluation and studies that fulfilled the exclusion criteria were eliminated. Key information was systematically organized into a structured Excel sheet, including study authors, publication year, biomarker values for IR (mean and standard deviation), participant numbers in both patient and control groups and total sample size. Recorded variables encompassed study design, biological sample type (serum, plasma, blood), duration since acute infection, intensive care unit admission during the initial illness, participant age, sex distribution and geographic setting. Discrepancies in data entry were addressed in consultation with the senior author (MM). The studies’ methodological quality was assessed using the Immunological Confounder Scale (ICS), first introduced by Andrés-Rodríguez et al. and later adapted by MM for IR research in LC (Ref. 32). The ICS consists of two complementary instruments as shown in Supplementary Table 3: the Quality Scale, which evaluates study design characteristics such as sample size, confounder control and sampling duration (scoring range 0–10, with higher scores indicating superior quality), and the redpoints scale, which assesses the degree of potential bias in LC immune activation research (scoring range 0–26, with lower scores reflecting greater methodological rigor). These instruments have been employed in prior meta-analyses related to immune activation and kynurenine pathway disruptions in LC, as well as in studies on lipid peroxidation and tryptophan metabolism in affective disorders (Refs 9, 33–35).

### Data analysis

In the present meta-analysis, we employed Comprehensive Meta-Analysis (CMA) software, version 4, in line with PRISMA recommendations (see Supplementary Table 4) to conduct all analyses. Biomarkers were included in the analysis only when at least two independent studies provided data. The Glycaemic–Insulinaemic Dysregulation index was calculated by combining insulin and fasting glucose under the dependency assumption and when both biomarkers reported by the same study. Likewise, the Insulin Secretion Axis Index was derived by integrating insulin and C-peptide values. Glucose–Insulin System Deviations and Integrated Glycaemic Burden were computed by combining glucose and insulin and glucose and HbA1c respectively.

Pooled effect sizes were computed using a random-effects model with constrained maximum likelihood estimation, with results expressed as standardized mean differences (SMDs) and 95% confidence intervals (CIs). A two-tailed *p* value <0.05 was considered statistically significant. Interpretation of effect sizes followed Cohen’s guidelines (Ref. 36), where SMD values of 0.20, 0.50 and 0.80 correspond to small, medium and large effects respectively. Heterogeneity was assessed through *τ^2^*, Q statistics and *I* ^2^, while meta-regression analyses were applied to examine moderators of variability. Robustness of results was tested using leave-one-out sensitivity analyses.

To evaluate publication bias, we applied the fail-safe *N* test, continuity-corrected Kendall’s tau and Egger’s regression intercept (one-tailed for the latter two). Where Egger’s test indicated asymmetry, the trim-and-fill method was employed to estimate missing studies and adjust pooled outcomes. The number of imputed studies indicate the extent of funnel plot asymmetry necessary to achieve symmetry and should be understood as a statistical correction rather than the detection of genuinely absent studies. Funnel plots were generated to visually examine the relationship between study precision and SMD, including both observed and imputed studies. In addition to Egger’s test, small-study effects were assessed using funnel plot symmetry and sensitivity analyses that removed studies with small sample sizes. Meta-regression analyses were performed to investigate potential sources of heterogeneity. Moderators including age, sex, latitude and post-COVID duration were individually incorporated into the regression models. Results were presented as *z* values alongside their respective one-tailed *p* values, derived from a random effects model framework. The certainty of evidence for each outcome was evaluated using the Grading of Recommendations Assessment, Development and Evaluation (GRADE) methodology (Ref. 37). Outcomes were assessed considering study limitations, consistency, directness, precision and potential publication bias and were categorized as high, moderate, low or very low certainty.

## Results

### Search outcomes

**Figure 1.**
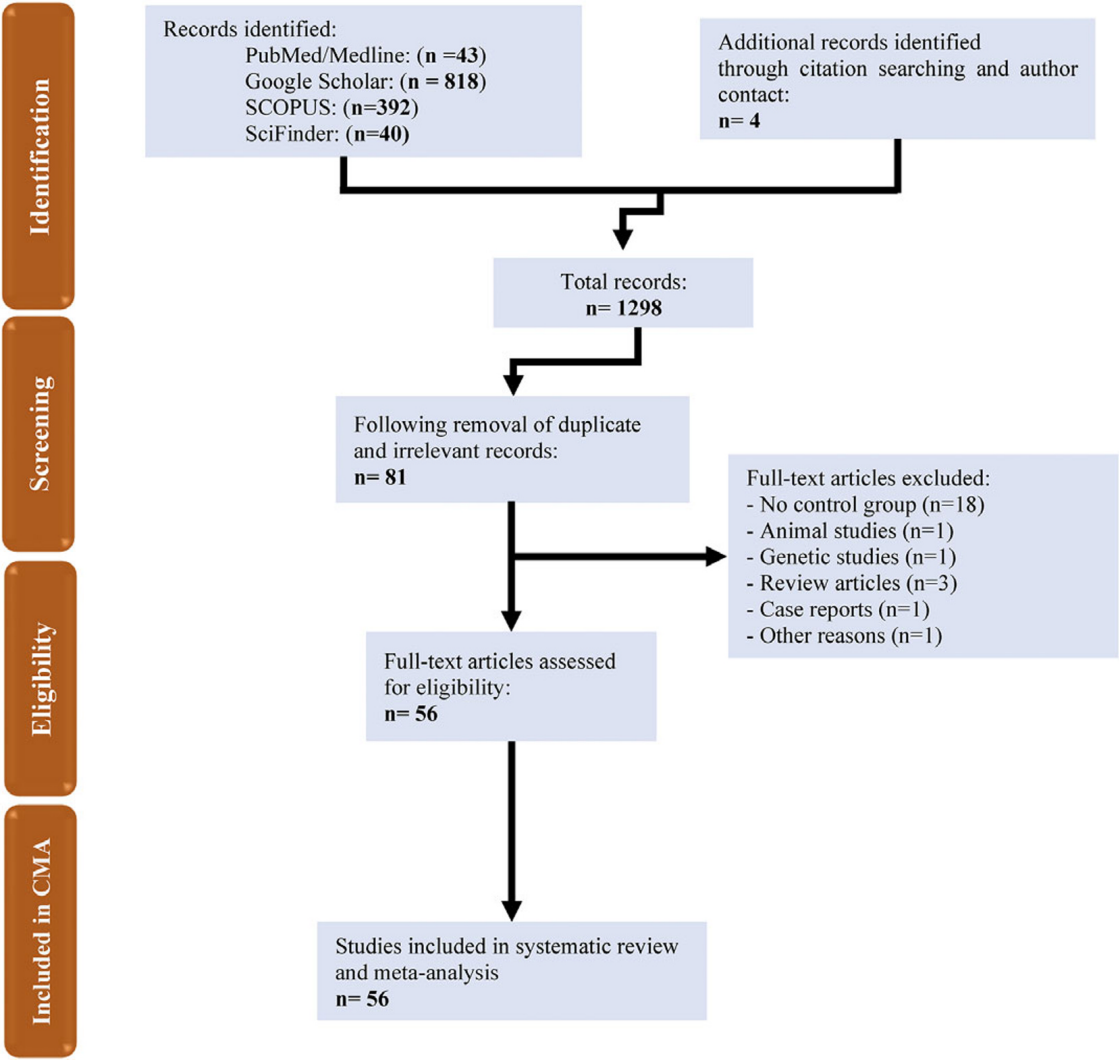
The PRISMA flow chart. Figure 1. long description.

Databases including PubMed, Google Scholar, SciFinder and SCOPUS were systematically searched using pre-specified keywords and MeSH terms (see Supplementary Table 2). As shown in the PRISMA flowchart (Figure 1), the initial search yielded 1253 articles. After removing duplicates and irrelevant studies, 81 remained for detailed screening. Of these, 56 met the inclusion criteria for the systematic review and 56 studies were ultimately included in the meta-analysis according to the pre-defined eligibility standards (Refs 7,17,18,24–26,38–66,67–93).

The meta-analysis comprised 9623 participants, including 5340 LC patients and 4283 control subjects, with ages ranging from 11 to 70 years. Most studies utilized routine laboratory tests alongside ELISA techniques to assess IR biomarkers. The included studies represented a wide geographic distribution, led by the United States (Ref. 18) studies, followed by Italy (Ref. 5). Iraq and Spain each contributed four studies, while Saudi Arabia contributed three studies. Brazil, Romania, Russia, Turkey and Ukraine each contributed two studies. Additional single-study contributions were from Australia, China, Germany, India, Indonesia, Iran, Japan, Malta, Oman, Poland, Slovakia and the United Kingdom. Study quality assessment showed median (min–max) values of 4 (min = 0, max = 9.5) for quality and 14.5 (min = 3.5, max = 26) for redpoints (Supplementary Table 5).

### Primary outcome variables

#### 3.2.1. HOMA-IR

**Figure 2.**
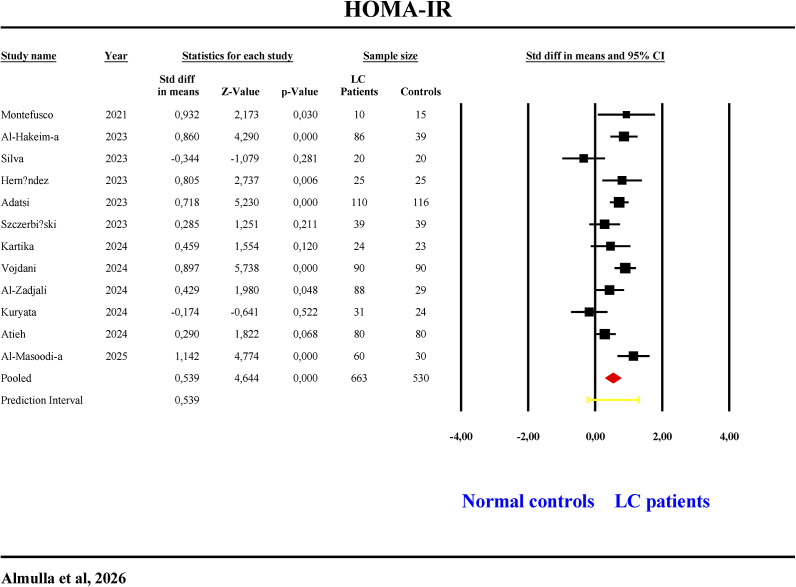
The forest plot of HOMA-IR in patients with long COVID (LC) and normal controls. Figure 2. long description.

Twelve studies assessed HOMA-IR (Table 1, Figure 2). None reported CIs entirely below zero, while seven had CIs entirely above. Five had overlapping CIs (two negative, three positive SMDs). LC patients exhibited significantly higher HOMA-IR values, with a moderate effect size (SMD = 0.539, 95% CI: 0.311–0.766, *p* < 0.001, *I* ^2^ = 69.11) (Table 2, Figure 2). No publication bias was found (Table 3).

**Table 2.** Results of meta-analysis performed on several outcomes (glucose homeostasis indices and biomarkers) variables with combined different media and separately Table 2. long description.

Outcome feature sets	Main statistics						Heterogeneity statistics						
	*n*	Groups	SMD	95% CI	*z*	*p*	PI	*Q*	df	*p*	*I^2^* *(%)*	*τ^2^*	*Τ*
HOMA-IR	12	Overall	0.539	0.311; 0.766	4.644	<0.001	−0.225;1.302	35.615	11	<0.001	69.114	0.104	0.322
Insulin	14	Overall	0.424	0.113; 0.735	2.673	0.008	−0.784;1.632	82.132	13	<0.001	84.172	0.531	0.282
Fasting blood glucose	12	Overall	0.734	0.190; 1.277	2.647	0.008	−1.430;2.898	261.726	11	<0.001	95.797	0.866	0.931
Random blood sugar	31	Overall	0.325	0.133; 0.517	3.324	0.001	−0.699;1.349	299.117	30	<0.001	89.970	0.241	0.491
HbA1c	16	Overall	0.189	−0.001; 0.378	1.953	0.051	−0.495;0.872	52.221	15	<0.001	71.276	0.092	0.304
HOMA-%B	5	Overall	0.827	−0.358; 2.012	1.367	0.171	−3.802;5.456	123.847	4	<0.001	96.770	1.750	1.323
HOMA-%S	2	Overall	−0.956	−1.256; −0.656	−6.238	<0.001	–	0.060	1	0.806	0.000	0.000	0.000
C-peptide	3	Overall	0.280	−0.095; 0.656	1.463	0.144	–	0.249	2	0.883	0.000	0.000	0.000
Glycaemic–Insulinaemic Dysregulation Index	7	Overall	0.600	−0.077; 1.277	1.737	0.082	−1.841;3.041	120.344	6	<0.001	95.014	0.783	0.885
Glucose–Insulin System Deviations	43	Overall	0.404	0.228; 0.579	4.507	<0.001	−0.697;1.504	470.344	42	<0.001	91.070	0.289	0.538
Insulin Secretion Axis Index	3	Overall	0.307	−0.071; 0.685	1.592	0.111	–	1.297	2	0.527	0	0	0
Integrated Glycaemic Burden	49	Overall	0.396	0.228;0.565	4.610	<0.001	−0.728;1.521	573.905	48	<0.001	91.636	0.305	0.552
Adiponectin	4	Overall	0.054	−0.182; 0.289	0.445	0.656	–	2.477	3	0.479	0.000	0.000	0.000
Leptin	5	Overall	0.165	−0.224; 0.554	0.832	0.406	−1.201;1.532	17.869	4	0.001	77.615	0.145	0.381
Ghrelin	3	Overall	0.223	−1.170; 1.165	0.313	0.754	–	29.916	2	<0.001	93.315	1.413	1.189

HOMA-IR: homeostatic model assessment of insulin resistance; HbA1c: glycated haemoglobin; HOMA-%B: homeostatic model assessment of beta-cell function; HOMA-%S: homeostatic model assessment of insulin sensitivity.

**Table 3.** Results on publication bias Table 3. long description.

Outcome feature sets	*p* (Kendall’s)	*p* (Egger’s)	Missing studies (side)	After adjusting
HOMA-IR	0.186	0.205	1 (Left)	–
Insulin	0.330	0.039	0	–
Fasting blood glucose	0.032	0.037	3 (Right)	1.040 (0.406;1.674)
Random blood glucose	0.138	0.116	4 (Right)	–
HbA1c	0.482	0.394	1 (Right)	–
HOMA-%B*	0.500	0.168	0	–
HOMA-%S*	0.500	0.405	0	–
C-peptide*	0.500	0.244	0	–
Glycaemic–Insulinaemic Dysregulation Index*	0.183	0.041	2 (Right)	0.946 (0.143;1.749)
Glucose–Insulin System Deviations	0.074	0.029	11 (Right)	0.698 (0.484;0.911)
Insulin Secretion Axis Index*	0.500	0.220	0	–
Integrated Glycaemic Burden	0.072	0.028	13 (Right)	0.693 (0.492;0.893)
Adiponectin*	0.367	0.313	1 (Left)	–
Leptin*	0.403	0.450	1 (Left)	0.083 (−0.277; 0.445)
Ghrelin*	0.148	0.0196	0	0

HOMA-IR: homeostatic model assessment of insulin resistance; HbA1c: glycated haemoglobin; HOMA-%B: homeostatic model assessment of beta-cell function; HOMA-%S: homeostatic model assessment of insulin sensitivity. * Publication-bias analyses for outcomes based on fewer than 10 studies should be interpreted cautiously because of limited statistical power.

#### Insulin

**Figure 3.**
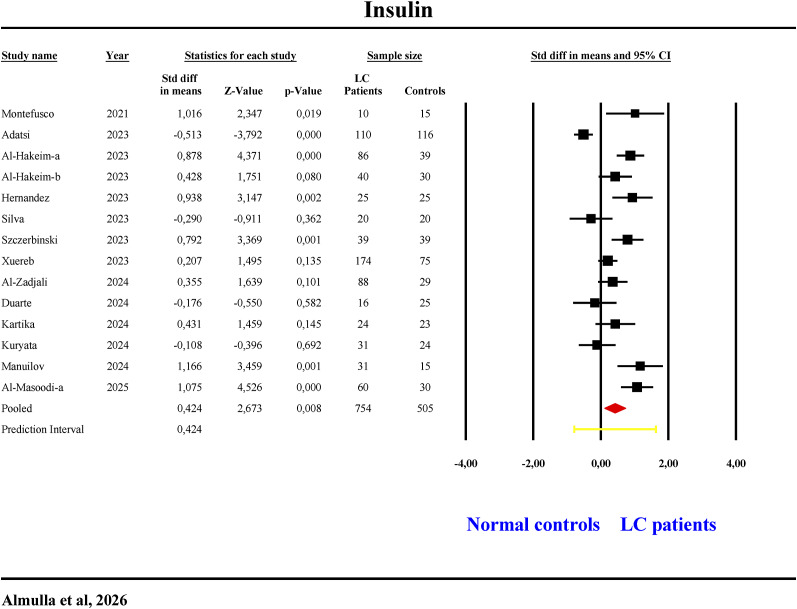
The forest plot of insulin in patients with long COVID (LC) and normal controls. Figure 3. long description.

Fourteen studies examined insulin levels (Table 1, Figure 3). One study reported CIs below zero, six above and seven overlapping (three negative, four positive SMDs). LC patients showed significantly increased (SMD = 0.424, 95% CI: 0.113–0.735, *p* = 0.008, *I* ^2^ = 84.17) insulin levels (Table 2, Figure 3). Egger’s and Kendall tests confirmed no publication bias (Table 3).

#### Fasting blood glucose

**Figure 4.**
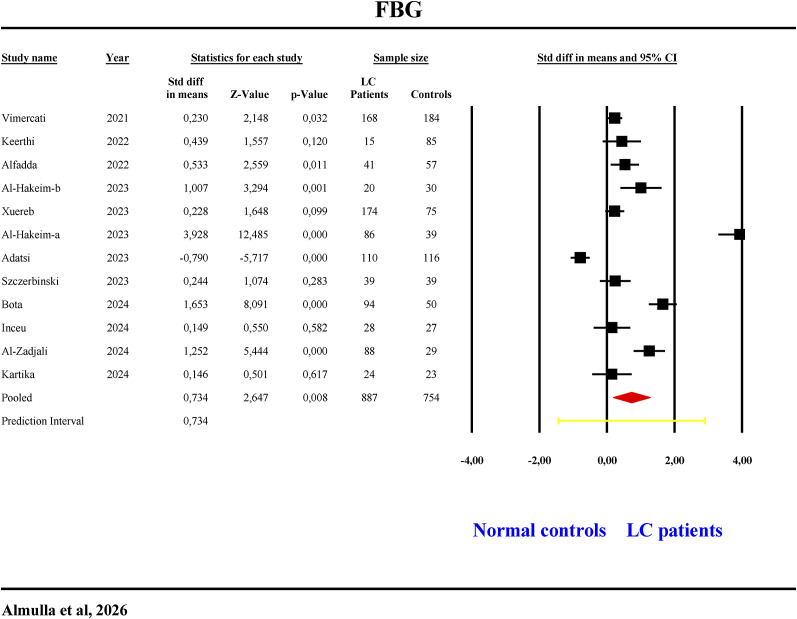
The forest plot of fasting blood glucose (FBG) in patients with long COVID (LC) and normal controls. Figure 4. long description.

Twelve studies assessed FBG (Table 1, Figure 4). One study had CIs below zero, five above and six overlapping with five positive SMDs. FBG levels were significantly elevated with a large effect size (SMD = 0.734, 95% CI: 0.190–1.277, *p* = 0.008, *I* ^2^ = 95.79) in LC patients (Table 2). Publication bias was found with three missing studies on the right side of the funnel plot and imputing them raised the effect size to 1.040 (Table 3).

#### Random blood sugar

**Figure 5.**
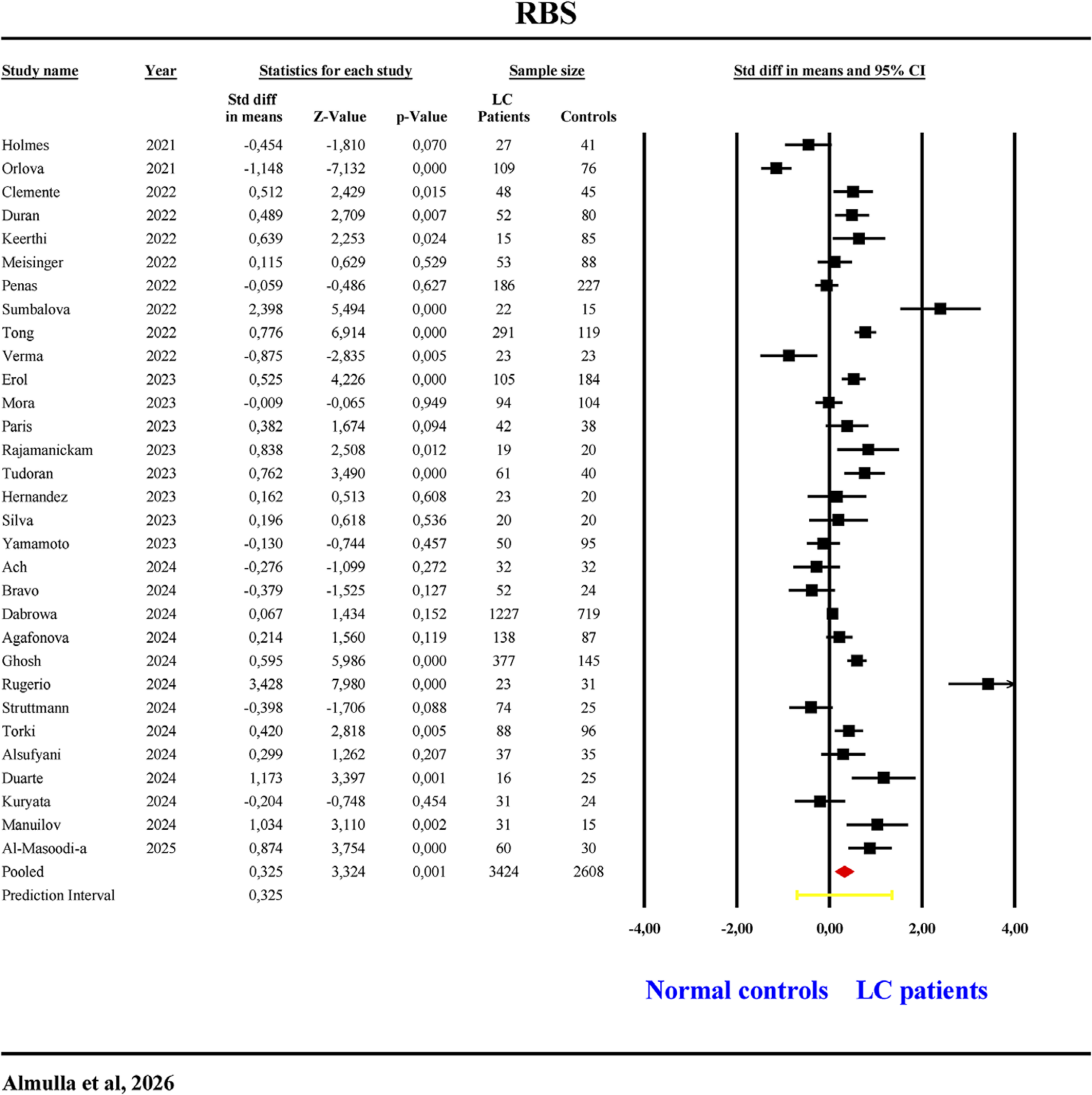
The forest plot of random blood sugar (RBS) in patients with long COVID (LC) and normal controls. Figure 5. long description.

Thirty-one studies examined RBS (Table 1, Figure 5). Two studies had CIs below zero, 14 above and 15 overlapping (eight negative, seven positive SMDs). LC patients exhibited significantly higher (SMD = 0.325, 95% CI: 0.133–0.517, *p* = 0.001, *I* ^2^ = 89.97) RBS levels (Table 2). Egger’s and Kendall tests indicated no publication bias (Table 3).

#### HbA1C

Sixteen studies investigated HbA1C (Table 1, Supplementary Figure 1). One study showed CIs below zero, six above and nine overlapping (three negative, six positive SMDs). LC patients demonstrated no significant change in (HbA1C levels (Table 2, Supplementary Figure 1), with no publication bias detected (Table 3).

#### HOMA-%B

In this study, data from five studies were combined to analyse HOMA-%B (Table 1, Supplementary Figure 2). Overall, LC patients demonstrated no significant change in HOMA-%B compared with normal controls (Table 1). Egger’s and Kendall tests confirmed the absence of publication bias (Table 3).

#### HOMA-%S

Two studies assessed HOMA-%S (Table 1, Supplementary Figure 3). Both studies showed CIs entirely below zero, indicating significantly reduced (SMD = −0.956, 95% CI: −1.256; −0.656, *p* < 0.001, *I^2^* = 0) HOMA-%S in LC patients (Table 2). No publication bias was detected (Table 3).

#### C peptide

Three studies evaluated C-peptide levels (Table 1). Table 2 indicates no significant difference in C-peptide concentrations between LC patients and normal controls.

### Secondary outcomes variables

#### Glycaemic–Insulinaemic Dysregulation Index

The meta-analysis included seven studies evaluating the composite of the Glycaemic–Insulinaemic Dysregulation Index (Table 1, Supplementary Figure 4). One study presented CIs entirely below zero, while four studies had CIs entirely above zero. Two studies showed overlapping CIs which both report positive SMDs. Overall, LC patients exhibited no significant alteration in this Glycaemic–Insulinaemic Dysregulation Index compared to controls (Table 2, Supplementary Figure 4). Nevertheless, publication bias detected two missing studies on the right side of the funnel plot and adjusting for these studies made the result significantly increased (Table 3).

#### Glucose–Insulin System Deviations

Forty-three studies examined the Glucose–Insulin System Deviations index (Table 1, Supplementary Figure 5). Three studies had CIs entirely below zero and 20 had CIs entirely above zero. Twenty showed overlapping CIs (9 negative, 11 positive SMDs). The index was significantly elevated (SMD = 0.404, 95% CI: 0.228–0.579, *p* < 0.001, *I^2^* = 91.07) in LC patients (Table 2, Supplementary Figure 5). Funnel plot analysis revealed publication bias, with 11 missing studies on the right side; adjustment increased the pooled SMD to 0.698 (Table 3).

#### Insulin Secretion Axis Index

This meta-analysis involves three studies examined the Insulin Secretion Axis Index (Table 1, Supplementary Figure 6). All of them showed overlapping CIs with positive SMDs. No significant change in Insulin Secretion Axis Index between LC patients and healthy controls. Table 3 indicated no publication bias.

#### Integrated Glycaemic Burden

Forty-nine studies examined the Integrated Glycaemic Burden (Table 1, Supplementary Figure 7). Three studies had CIs entirely below zero and 23 had CIs entirely above zero. Twenty-three showed overlapping CIs (9 negative, 14 positive SMDs). The composite was significantly elevated (SMD = 0.396, 95% CI: 0.228–0.565, *p* < 0.001, *I* ^2^ = 91.63) in LC patients (Table 2, Supplementary Figure 7). Funnel plot analysis revealed publication bias, with 13 missing studies on the right side; adjustment increased the pooled SMD to 0.693 (Table 3).

#### Adiponectin

Four studies assessed adiponectin levels (Table 1). Table 2 showed no significant difference in adiponectin concentrations between LC patients and normal controls.

#### Leptin

Five studies assessed leptin levels (Table 1). Table 2 revealed no significant difference in leptin concentrations between LC patients and normal controls.

#### Ghrelin

Three studies evaluated ghrelin levels (Table 1). Table 2 indicated no significant difference in ghrelin concentrations between LC patients and normal controls.

### Meta-regression analysis

Meta-regression analysis explored sources of heterogeneity among studies investigating IR in LC (Supplementary Table 6). A post-acute period exceeding 6 months – the phase during which post-COVID manifestations emerge – had a significant effect (*p* < 0.05). Similarly, medication use significantly influenced most outcomes.

### Certainty of evidence

The GRADE assessment revealed that the certainty of evidence varied from low to very low for the outcomes of the current study, as shown in Supplementary Table 7. HOMA-IR, fasting insulin, FBG, Glycaemic–Insulinaemic Dysregulation Index and Insulin Secretion Axis Index were supported by low-certainty evidence, primarily reflecting the observational design of the included studies and substantial between-study heterogeneity observed across several analyses. Outcomes exhibiting significant heterogeneity, imprecision or insufficient data were assigned very low certainty, especially regarding Glucose–Insulin System Deviations, Integrated Glycaemic Burden and adipokine measures. Sensitivity analyses (Supplementary Figures 8–18) confirmed the robustness of all pooled estimates, with no evidence of small-study effects.

## Discussion

### Persistent insulin resistance and hyperglycaemia in long COVID

This meta-analysis is, to our knowledge, the first to comprehensively examine IR indices and related biomarkers in LC compared with normal controls. Our first major finding is that LC is associated with elevated IR and hyperglycaemia indicated by elevated HOMA-IR, insulin, FBG and RBS, alongside reduced HOMA-%S. Furthermore, newly constructed indices such as Glycaemic-Insulinaemic Dysregulation Index, Glucose–Insulin System Deviations and Integrated Glycaemic Burden were also increased in LC patients emphasizing the consequent IR and hyperglycaemia after acute COVID infection. Together, these results indicate a phenotype marked by hyperglycaemia, compensatory up-regulation of insulin secretion and reduced tissue sensitivity to insulin.

The current findings are in line with prior evidence demonstrating increased IR in LC (Refs 7, 25, 27, 66, 94) and suggest that COVID-19 may increase diabetes risk by aggravating IR (Ref. 74). COVID-19-related metabolic dysfunction might clinically manifest as new-onset diabetes, asymptomatic IR and glucose intolerance (Ref. 24). Man et al. demonstrated a markedly increased risk of IR and diabetes, reporting that up to 75% of patients with long COVID developed diabetes within 1 year of infection (Ref. 95).

Various mechanisms may contribute to IR and hyperglycaemia in LC. Notably, LC-related IR was predicted by elevated peak body temperature during acute illness, suggesting the role of acute-phase inflammatory severity in subsequent metabolic outcomes including insulin resistance (Ref. 7). In general, viral infections may induce IR; in murine models, IFN-γ might down-regulate muscle insulin receptors, leading to compensatory hyperinsulinaemia (Ref. 96). SARS-CoV-2 invasion of pancreatic β cells and other metabolic tissues can disrupt insulin production and signalling (Refs 20, 97). Virus-induced metabolic reprogramming towards anaerobic respiration, accompanied by acid–base disturbances, may exacerbate IR (Ref. 98). SARS-CoV-2 infection of adipose tissue and β cells promotes adipose dysfunction (e.g. reduced adiponectin) and β-cell impairment, both contributing to IR (Ref. 99). SARS-CoV-2 inhibits peroxisome proliferator-activated receptor-gamma co-activator 1-alpha (PGC-1α) and irisin, leading to compromised mitochondrial function and energy metabolism, which results in decreased glucose uptake and increased IR (Refs 97, 100).

In LC, elevated cytokines may drive serine phosphorylation of insulin receptor substrate-1(IRS-1) which impairs insulin signalling and glucose uptake (Refs 9, 15). Ongoing immune activation, including increased NETosis and altered microRNAs such as miR-21-5p, has been linked to post-COVID IR (Ref. 101). Sustained inflammatory, oxidative and nitrosative stress signalling – such as activation of NF-κB and cytokines – can disrupt insulin receptor pathways (Refs 102, 103). However, Al-Hakeim et al. reported that, in LC, inflammation during the acute phase exerts only a minor effect on new-onset IR, whereas activated IO&NS pathways during the chronic phase appear to have no significant impact (Ref. 7).

### Pancreatic β cell and adipokine levels in LC disease

Our second major findings show no significant alteration in HOMA-%B adiponectin, ghrelin or leptin in LC patients versus normal controls. These results point to normal β-cell function and an abnormal glucose metabolism beyond the β cells. However, evidence supporting β-cell dysfunction in LC exists (Ref. 81), but the literature is mixed: some studies reported no significant change in HOMA-%B aligning with our findings (Refs 7, 90), whereas others observed increased HOMA-%B (Refs 24, 104). These discrepancies likely reflect heterogeneity in disease stage, acute-phase severity, treatment exposures and recovery trajectories.

SARS-CoV-2 infects β cells through ACE2, leading to direct cytopathic effects and reduced insulin secretion (Ref. 105). LC exhibits significant immune activation characterized by elevated levels of cytokines, chemokines and growth factors, which may intensify β-cell stress and contribute to cell loss (Refs 9, 106). Cytokines, including IFN-γ, have the capacity to inhibit insulin gene expression and β-cell functionality (Ref. 107). Autoimmune mechanisms may also contribute; LC is associated with increased autoantibodies targeting self-proteins (Refs 8, 94), consistent with post-viral immune dysregulation that could involve islet antigens (Ref. 108).

Molecular mimicry presents an intriguing mechanism for the rapid onset of islet autoimmunity. Viral epitopes that share structural characteristics with islet antigens may enhance pre-existing autoimmune tendencies, accelerating the onset of T1DM instead of initiating autoimmunity from the beginning (Ref. 109). Extended β-cell infection may enhance MHC-I expression, thereby maintaining antigen presentation and facilitating epitope spreading as further islet antigens are revealed (Ref. 110). This cascade promotes the activation of cytotoxic CD8+ T cells and the diversification of autoantibody responses to insulin, GAD and IA-2, leading to progressive β-cell loss and hyperglycaemia (Ref. 111). Increased NET formation during COVID-19 may exacerbate autoimmune inflammation and adjacent tissue damage, thereby compromising islet integrity (Ref. 112). All in all, these findings suggest a trajectory in which inflammation, oxidative stress and potential autoimmunity intersect with glucose metabolism in LC without direct impact on the pancreatic β-cells functions.

### LC disease predisposes patients to diabetes mellitus and its consequences

Epidemiologic data indicates increased risk after COVID-19 for new-onset diabetes (types 1 and 2), metabolic syndrome and related complications (Refs 113, 114). Risk peaks in the first 3 months post-infection yet remains elevated over the longer term (Refs 113, 115). Research indicates that this risk may be partially independent of conventional factors like BMI, suggesting a direct impact from COVID-19 itself (Refs 20, 113). Indeed, in our meta-regression analysis we did not find any impact for BMI on the outcomes. These observations are consistent with our aggregated evidence of persistent IR and β-cell dysfunction in LC, as well as the mechanistic connections to inflammation, oxidative stress, endothelial dysfunction and possible autoimmunity outlined previously.

The clinical implications are evident. Individuals recovering from COVID-19, especially those with LC features, may require metabolic follow-up to detect emergent IR, dysglycaemia, dyslipidaemia and related metabolic aberrations, including hepatic involvement (Ref. 10). The convergence of adverse lipid profiles, insulin signalling defects and β-cell stress argues for integrated management strategies that address immune activation, oxidative pathways and metabolic regulation simultaneously. While definitive interventional trials in LC remain limited, the mechanistic and epidemiologic signals justify proactive monitoring and targeted risk modification.

While various biological mechanisms were reported as potential contributors to metabolic dysfunction in LC, the robustness of evidence varies significantly among paths. Although sustained immune-inflammatory response and oxidative and nitrosative stress have been directly linked to the LC disease (Refs 9, 13), β-cell injury mediated by ACE2, NETosis, endothelial dysfunction and molecular mimicry is derived largely from acute COVID-19 studies or indirect observations (Refs 116–120). Consequently, certain postulated pathways should presently be considered physiologically viable hypotheses necessitating additional validation in specialized long COVID research.

It is crucial to contemplate alternative interpretations that IR and the associated metabolic anomalies are recognized as risk factors for severe COVID-19 and may heighten susceptibility to enduring post-acute symptoms, suggesting the potential for reverse causation (Refs 113, 121, 122). Furthermore, residual confounding from pre-existing metabolic diseases, obesity and corticosteroid exposure during acute infection cannot be entirely ruled out (Refs 123–125), while certain studies have associated corticosteroid use during the acute phase with a reduced incidence of LC (Ref. 126). The current investigation corroborates previous findings indicating a significant risk of new-onset diabetes and enduring disruptions in glucose metabolism subsequent to SARS-CoV-2 infection (Refs 113, 114). Future prospective studies incorporating pre-infection metabolic evaluations are essential for more accurately determining causality.

All in all, this integrated perspective underscores the necessity for multidisciplinary LC care pathways and encourages additional research to enhance phenotyping, identify modifiable targets and evaluate interventions that influence neuro-immune–metabolic–oxidative (NIMETOX) pathways-related processes (Ref. 127).

## Limitations

Although most results of this meta-analysis were not affected by publication bias, several limitations should be acknowledged. First, the analysis was constrained by the limited data available in the included studies, which prevented assessment of the effects of treatments administered during the acute phase or post-recovery period and vaccination state. Future research should investigate how therapeutic interventions during these stages influence LC outcomes. Second, the study would have been strengthened by the inclusion of additional components of the immune–metabolic–oxidative stress pathways (Ref. 127). Future investigations should aim to examine multiple related biomarkers simultaneously to better characterize their contribution to the pathophysiology of LC. HOMA-%S finding should be interpreted cautiously because it was derived from only two independent studies from the same research group. Additional studies from independent populations are required to confirm this observation.

Third, due to the limited number of studies reporting pre-existing diabetes, metabolic syndrome and obesity data, it may be challenging to determine whether LC is associated with the new onset of T2DM. Nevertheless, some rigorously controlled studies have demonstrated that SARS-CoV-2 infection is accompanied by de novo T2DM some months after the acute infectious phase (Refs 7, 27). Subsequent studies should provide detailed information on co-morbid conditions such as obesity, metabolic syndrome and related factors to enable a more comprehensive understanding of their role in LC-associated IR. A few secondary outcomes were derived from a limited number of studies (fewer than five). Consequently, the pooled results must be interpreted cautiously, as their reliability hinges on the availability of further primary studies in subsequent research. The included studies employed surrogate biomarkers like HOMA-IR instead of gold-standard assessments of insulin sensitivity, such as hyperinsulinaemic-euglycaemic clamp methods. Consequently, the mechanistic interpretations remain speculative and should be regarded as possible biological explanations rather than definitive evidence of causality. Finally, although we employed several databases and different search methods, Embase was not accessed due to the absence of institutional access, probably restricting the identification of further eligible research. This meta-analysis involved several exploratory analyses and meta-regression; thus, some findings should be interpreted cautiously and should be confirmed independently in future studies.

## Conclusion

**Figure 6.**
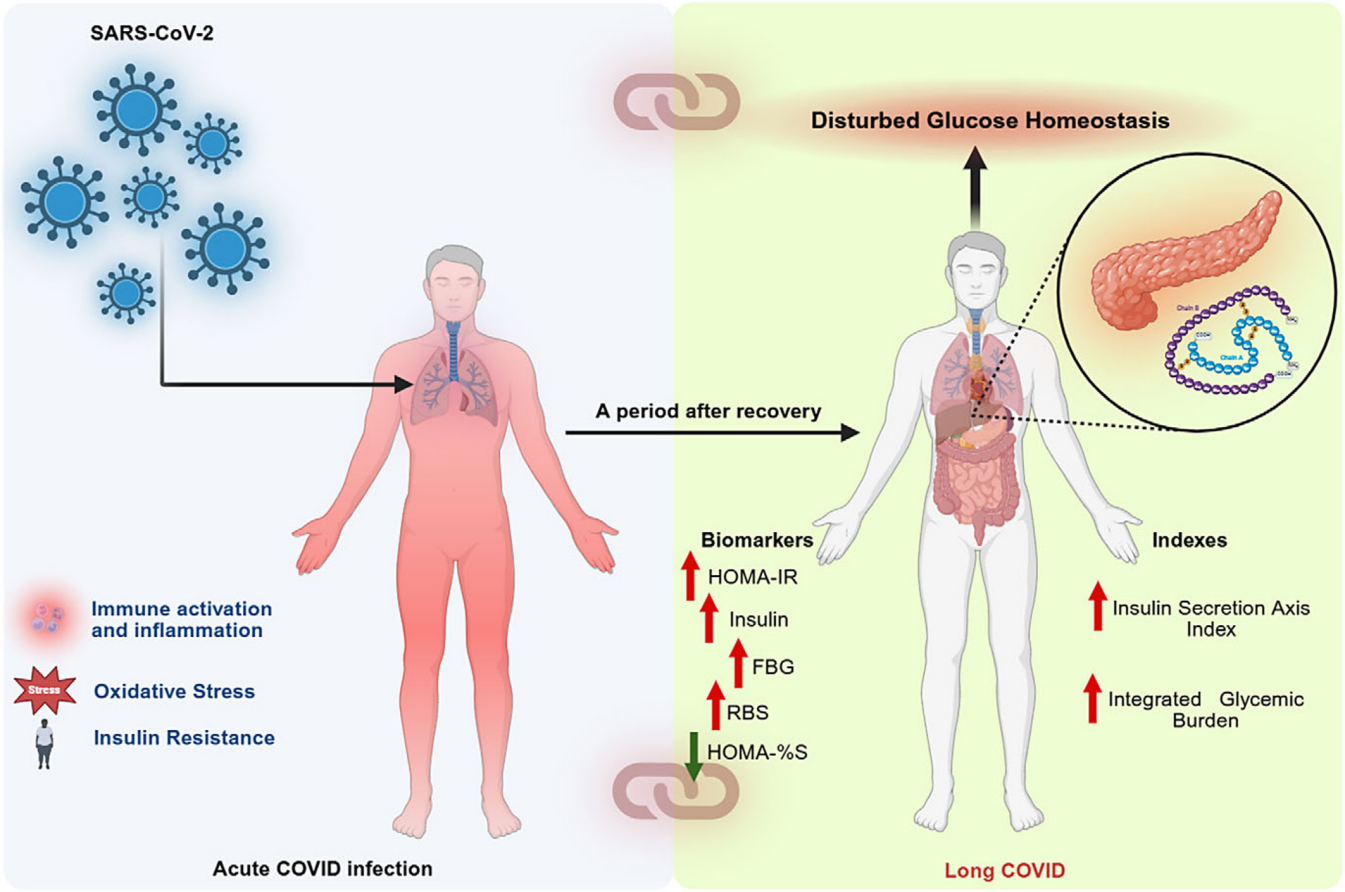
Summary of the insulin resistance (IR) in long COVID (LC) disease. SARS-CoV-2: severe acute respiratory syndrome coronavirus 2; HOMA-IR: homeostatic model assessment for insulin resistance; FBG: fasting blood glucose, HbA1c: haemoglobin A1C; RBS: random blood sugar; HOMA-%B: homeostasis model assessment for beta-cell function; HOMA-%S: homeostatic model assessment for insulin sensitivity. Figure 6. long description.

The current meta-analysis, which is the first comprehensive evidence, suggests that LC disease is characterized by persistent IR and disturbances in glucose homeostasis, particularly elevated HOMA-IR, fasting insulin and fasting blood glucose levels (Figure 6) reflecting sustained metabolic disruptions beyond the acute infection phase. Our findings highlight the clinical significance of regular metabolic monitoring in populations affected by LC. Future longitudinal and interventional studies are necessary to assess the persistence, clinical significance and modifiability of IR in this condition.

## Supporting information

10.1017/erm.2026.10064.sm001 Almulla et al. supplementary material Almulla et al. supplementary material

## Data Availability

The corresponding author (MM) will evaluate reasonable requests for access to the dataset (Excel file) used in this meta-analysis, after all contributing authors have completed their data usage.

## Supplementary Material

The supplementary material for this article can be found at http://doi.org/10.1017/erm.2026.10064.

## Long descriptions

### Table 1. Long description

The table contains 15 outcome profiles with the following data columns: n studies, Side of 95 percent confidence intervals (less than 0, Overlap 0 and S M D less than 0, Overlap 0 and S M D greater than 0, greater than 0), Patient cases, Control cases, and Total number of participants.

* H O M A minus I R: 12 studies, 7 with S M D greater than 0. 663 patients, 530 controls, 1193 total.

* Insulin: 14 studies, 6 with S M D greater than 0. 754 patients, 505 controls, 1259 total.

* Fasting blood glucose: 12 studies, 5 with S M D greater than 0 and 5 overlapping 0 with S M D greater than 0. 887 patients, 754 controls, 1641 total.

* Random blood glucose: 31 studies, 14 with S M D greater than 0. 3424 patients, 2608 controls, 6032 total.

* H b A 1 c: 16 studies, 6 with S M D greater than 0 and 6 overlapping 0 with S M D greater than 0. 1056 patients, 953 controls, 2009 total.

* H O M A minus percent B: 5 studies, 2 with S M D greater than 0. 269 patients, 232 controls, 501 total.

* H O M A minus percent S: 2 studies, both with S M D less than 0. 146 patients, 69 controls, 215 total.

* C-peptide: 3 studies, all 3 overlapping 0 with S M D greater than 0. 109 patients, 306 controls, 415 total.

* Glycaemic-Insulinaemic Dysregulation index: 7 studies, 4 with S M D greater than 0. 551 patients, 351 controls, 902 total.

* Glucose-Insulin System Deviations: 43 studies, 20 with S M D greater than 0. 4316 patients, 3293 controls, 7609 total.

* Insulin Secretion Axis Index: 3 studies, all 3 overlapping 0 with S M D greater than 0. 50 patients, 63 controls, 113 total.

* Integrated Glycaemic Burden: 49 studies, 23 with S M D greater than 0. 4779 patients, 3830 controls, 8609 total.

* Adiponectin: 4 studies, 3 overlapping 0 with S M D greater than 0. 125 patients, 166 controls, 291 total.

* Leptin: 5 studies, 3 overlapping 0 with S M D greater than 0. 390 patients, 286 controls, 676 total.

* Ghrelin: 3 studies, 1 with S M D greater than 0 and 1 overlapping 0 with S M D greater than 0. 78 patients, 62 controls, 140 total.

Navigate back to Table 1..

### Table 2. Long description

The table is divided into two primary sections: Main statistics (n, Groups, S M D, 95% C I, z, p) and Heterogeneity statistics (P I, Q, df, p, I super 2, tau super 2, tau).

Key findings include:

* H O M A - I R: n = 12, S M D = 0.539, p < 0.001, I super 2 = 69.114%.

* Insulin: n = 14, S M D = 0.424, p = 0.008, I super 2 = 84.172%.

* Fasting blood glucose: n = 12, S M D = 0.734, p = 0.008, I super 2 = 95.797%.

* Random blood sugar: n = 31, S M D = 0.325, p = 0.001, I super 2 = 89.970%.

* HbA1c: n = 16, S M D = 0.189, p = 0.051, I super 2 = 71.276%.

* H O M A - % B: n = 5, S M D = 0.827, p = 0.171, I super 2 = 96.770%.

* H O M A - % S: n = 2, S M D = -0.956, p < 0.001, I super 2 = 0.000%.

* C-peptide: n = 3, S M D = 0.280, p = 0.144, I super 2 = 0.000%.

* Glycaemic-Insulinaemic Dysregulation Index: n = 7, S M D = 0.600, p = 0.082, I super 2 = 95.014%.

* Glucose-Insulin System Deviations: n = 43, S M D = 0.404, p < 0.001, I super 2 = 91.070%.

* Insulin Secretion Axis Index: n = 3, S M D = 0.307, p = 0.111, I super 2 = 0%.

* Integrated Glycaemic Burden: n = 49, S M D = 0.396, p < 0.001, I super 2 = 91.636%.

* Adiponectin: n = 4, S M D = 0.054, p = 0.656, I super 2 = 0.000%.

* Leptin: n = 5, S M D = 0.165, p = 0.406, I super 2 = 77.615%.

* Ghrelin: n = 3, S M D = 0.223, p = 0.754, I super 2 = 93.315%.

Navigate back to Table 2..

### Table 3. Long description

The table contains 15 rows of data across 5 columns.

* H O M A minus I R: p Kendall’s 0.186, p Egger’s 0.205, 1 missing study (Left), After adjusting: dash.

* Insulin: p Kendall’s 0.330, p Egger’s 0.039, 0 missing studies, After adjusting: dash.

* Fasting blood glucose: p Kendall’s 0.032, p Egger’s 0.037, 3 missing studies (Right), After adjusting: 1.040 (0.406; 1.674).

* Random blood glucose: p Kendall’s 0.138, p Egger’s 0.116, 4 missing studies (Right), After adjusting: dash.

* H b A 1 c: p Kendall’s 0.482, p Egger’s 0.394, 1 missing study (Right), After adjusting: dash.

* H O M A minus percent B: p Kendall’s 0.500, p Egger’s 0.168, 0 missing studies, After adjusting: dash.

* H O M A minus percent S: p Kendall’s 0.500, p Egger’s 0.405, 0 missing studies, After adjusting: dash.

* C minus peptide: p Kendall’s 0.500, p Egger’s 0.244, 0 missing studies, After adjusting: dash.

* Glycaemic-Insulinaemic Dysregulation Index: p Kendall’s 0.183, p Egger’s 0.041, 2 missing studies (Right), After adjusting: 0.946 (0.143; 1.749).

* Glucose-Insulin System Deviations: p Kendall’s 0.074, p Egger’s 0.029, 11 missing studies (Right), After adjusting: 0.698 (0.484; 0.911).

* Insulin Secretion Axis Index: p Kendall’s 0.500, p Egger’s 0.220, 0 missing studies, After adjusting: dash.

* Integrated Glycaemic Burden: p Kendall’s 0.072, p Egger’s 0.028, 13 missing studies (Right), After adjusting: 0.693 (0.492; 0.893).

* Adiponectin: p Kendall’s 0.367, p Egger’s 0.313, 1 missing study (Left), After adjusting: dash.

* Leptin: p Kendall’s 0.403, p Egger’s 0.450, 1 missing study (Left), After adjusting: 0.083 (minus 0.277; 0.445).

* Ghrelin: p Kendall’s 0.148, p Egger’s 0.0196, 0 missing studies, After adjusting: 0.

Navigate back to Table 3..

### Figure 1. Long description

A vertical sidebar on the left labels four phases: Identification, Screening, Eligibility, and Included in C M A.

1. Identification Phase: At the top left, a box titled Records identified lists PubMed/Medline n = 43, Google Scholar n = 818, S C O P U S n = 392, and SciFinder n = 40. At the top right, a box titled Additional records identified through citation searching and author contact lists n = 4. Arrows from both boxes merge into a central box.

2. Screening Phase: The merged flow leads to a box titled Total records n = 1298. An arrow points down to a box titled Following removal of duplicate and irrelevant records n = 81.

3. Eligibility Phase: An arrow points down to a box titled Full-text articles assessed for eligibility n = 56. A horizontal arrow points right to a box titled Full-text articles excluded, which lists: No control group n = 18, Animal studies n = 1, Genetic studies n = 1, Review articles n = 3, Case reports n = 1, and Other reasons n = 1.

4. Inclusion Phase: A final arrow points down to the bottom box titled Studies included in systematic review and meta-analysis n = 56.

Navigate back to Figure 1..

### Figure 2. Long description

A forest plot titled H O M A - I R. The table columns from left to right are Study name, Year, Statistics for each study (Std diff in means, Z-Value, p-Value), Sample size (L C Patients, Controls), and Std diff in means and 95% C I.

Twelve individual studies are listed from 2021 to 2025:

- Montefusco (2021): 0.932

- Al-Hakeim-a (2023): 0.860

- Silva (2023): -0.344

- Hernandez (2023): 0.805

- Adatsi (2023): 0.718

- Szczerbi?ski (2023): 0.285

- Kartika (2024): 0.459

- Vojdani (2024): 0.897

- Al-Zadjali (2024): 0.429

- Kuryata (2024): -0.174

- Atieh (2024): 0.290

- Al-Masoodi-a (2025): 1.142

The pooled analysis shows a standard difference in means of 0.539 with a Z-Value of 4.644 and a p-Value of 0.000. The total sample size is 663 L C patients and 530 controls.

The graphical forest plot on the right features an X-axis ranging from -4.00 to 4.00. A vertical line at 0.00 represents the null effect. Most studies show a square marker to the right of the zero line, indicating higher H O M A - I R in L C patients. A red diamond at the bottom represents the pooled effect, centered at 0.539. A yellow horizontal line below the diamond represents the prediction interval.

Navigate back to Figure 2..

### Figure 3. Long description

A forest plot titled Insulin. The table contains columns for Study name, Year, Statistics for each study (Std diff in means, Z-Value, p-Value), Sample size (L C Patients, Controls), and a visual representation of Std diff in means and 95% C I.

Individual study data:

* Montefusco (2021): 1.016, Z 2.347, p 0.019, L C 10, Controls 15.

* Adatsi (2023): -0.513, Z -3.792, p 0.000, L C 110, Controls 116.

* Al-Hakeim-a (2023): 0.878, Z 4.371, p 0.000, L C 86, Controls 39.

* Al-Hakeim-b (2023): 0.428, Z 1.751, p 0.080, L C 40, Controls 30.

* Hernandez (2023): 0.938, Z 3.147, p 0.002, L C 25, Controls 25.

* Silva (2023): -0.290, Z -0.911, p 0.362, L C 20, Controls 20.

* Szczerbinski (2023): 0.792, Z 3.369, p 0.001, L C 39, Controls 39.

* Xuereb (2023): 0.207, Z 1.495, p 0.135, L C 174, Controls 75.

* Al-Zadjali (2024): 0.355, Z 1.639, p 0.101, L C 88, Controls 29.

* Duarte (2024): -0.176, Z -0.550, p 0.582, L C 16, Controls 25.

* Kartika (2024): 0.431, Z 1.459, p 0.145, L C 24, Controls 23.

* Kuryata (2024): -0.108, Z -0.396, p 0.692, L C 31, Controls 24.

* Manuilov (2024): 1.166, Z 3.459, p 0.001, L C 31, Controls 15.

* Al-Masoodi-a (2025): 1.075, Z 4.526, p 0.000, L C 60, Controls 30.

Summary data:

* Pooled: 0.424, Z 2.673, p 0.008, Total L C 754, Total Controls 505. Represented by a red diamond centered to the right of the zero line.

* Prediction Interval: 0.424, represented by a yellow horizontal bar.

The X-axis for the plot ranges from -4.00 to 4.00. Values to the left of 0.00 favor Normal controls, and values to the right favor L C patients.

Navigate back to Figure 3..

### Figure 4. Long description

A forest plot titled F B G at the top. The table columns from left to right are Study name, Year, Statistics for each study (Std diff in means, Z-Value, p-Value), Sample size (L C Patients, Controls), and Std diff in means and 95% C I.

Twelve studies are listed from 2021 to 2024.

1. Vimercati (2021): 0,230 mean diff; 168 L C, 184 controls.

2. Keerthi (2022): 0,439 mean diff; 15 L C, 85 controls.

3. Alfadda (2022): 0,533 mean diff; 41 L C, 57 controls.

4. Al-Hakeim-b (2023): 1,007 mean diff; 20 L C, 30 controls.

5. Xuereb (2023): 0,228 mean diff; 174 L C, 75 controls.

6. Al-Hakeim-a (2023): 3,928 mean diff; 86 L C, 39 controls.

7. Adatsi (2023): -0,790 mean diff; 110 L C, 116 controls.

8. Szczerbinski (2023): 0,244 mean diff; 39 L C, 39 controls.

9. Bota (2024): 1,653 mean diff; 94 L C, 50 controls.

10. Inceu (2024): 0,149 mean diff; 28 L C, 27 controls.

11. Al-Zadjali (2024): 1,252 mean diff; 88 L C, 29 controls.

12. Kartika (2024): 0,146 mean diff; 24 L C, 23 controls.

The Pooled result shows a mean difference of 0,734 with a Z-Value of 2,647 and p-Value of 0,008, based on a total sample of 887 L C patients and 754 controls.

The forest plot on the right features a vertical line at 0,00 representing no effect. Data points are black squares with horizontal error bars. Most points fall to the right of the zero line, indicating higher F B G in L C patients. A red diamond at the bottom represents the pooled effect size of 0,734. A yellow horizontal line at the very bottom represents the Prediction Interval. The X-axis at the bottom is labeled from -4,00 to 4,00, with ‘Normal controls’ on the left and ‘L C patients’ on the right.

Navigate back to Figure 4..

### Figure 5. Long description

A forest plot titled R B S at the top. The table contains six main columns.

1. Study name and Year: Lists 30 individual studies including Holmes 2021, Orlova 2021, Clemente 2022, through Al-Masoodi-a 2025.

2. Statistics for each study: Includes Std diff in means, Z-Value, and p-Value.

3. Sample size: Divided into L C Patients and Controls.

4. Std diff in means and 95% C I: A graphical forest plot.

Data highlights:

* Individual study standardized difference in means ranges from -1,148 (Orlova) to 3,428 (Rugerio).

* The total sample size is 3424 L C patients and 2608 controls.

* The Pooled result shows a standardized difference in means of 0,325 with a Z-Value of 3,324 and a p-Value of 0,001.

* The forest plot on the right features a vertical line at 0,00 representing no effect. Most studies and the final pooled diamond are positioned to the right of the zero line, indicating higher R B S in L C patients.

* A red diamond at the bottom represents the pooled effect size.

* A yellow horizontal bar represents the Prediction Interval.

* The x-axis for the plot ranges from -4,00 to 4,00.

* Labels at the bottom right indicate Normal controls on the left of the zero line and L C patients on the right.

Navigate back to Figure 5..

### Figure 6. Long description

The diagram is divided into two vertical panels connected by a central arrow labeled A period after recovery.

Left Panel: Acute C O V I D infection.

At the top left, blue S A R S dash C o V dash 2 virions point an arrow toward the lungs of a red-shaded human silhouette. Below this, three icons with text describe the state: Immune activation and inflammation, Oxidative Stress, and Insulin Resistance.

Right Panel: Long C O V I D.

A white human silhouette is shown with internal organs visible. An arrow points from the head of this figure to a top banner labeled Disturbed Glucose Homeostasis. A circular inset zoom shows the pancreas and a molecular model of insulin with Chain A and Chain B.

Below the figure are two lists of clinical data:

1. Biomarkers: Red upward arrows indicate increases in H O M A dash I R, Insulin, F B G, and R B S. A green downward arrow indicates a decrease in H O M A dash percent S.

2. Indexes: Red upward arrows indicate increases in Insulin Secretion Axis Index and Integrated Glycemic Burden.

The two panels are further linked by chain link icons at the top and bottom of the dividing line.

Navigate back to Figure 6..
